# Effects of pH alterations on stress- and aging-induced protein phase separation

**DOI:** 10.1007/s00018-022-04393-0

**Published:** 2022-06-24

**Authors:** Xuejiao Jin, Min Zhou, Shuxin Chen, Danqi Li, Xiuling Cao, Beidong Liu

**Affiliations:** 1 State Key Laboratory of Subtropical Silviculture, School of Forestry and Biotechnology, Zhejiang A&F University, Lin’an, Hangzhou, 311300 China; 2 Department of Chemistry and Molecular Biology, University of Gothenburg, Medicinaregatan 9C, 413 90 Goteborg, Sweden; 3 Center for Large-Scale Cell-Based Screening, Faculty of Science, University of Gothenburg, Medicinaregatan 9C, 413 90 Goteborg, Sweden

**Keywords:** Acidification, Membrane-less compartment, Neurodegenerative disease, Tumorigenesis, Protein aggregation

## Abstract

Upon stress challenges, proteins/RNAs undergo liquid–liquid phase separation (LLPS) to fine-tune cell physiology and metabolism to help cells adapt to adverse environments. The formation of LLPS has been recently linked with intracellular pH, and maintaining proper intracellular pH homeostasis is known to be essential for the survival of organisms. However, organisms are constantly exposed to diverse stresses, which are accompanied by alterations in the intracellular pH. Aging processes and human diseases are also intimately linked with intracellular pH alterations. In this review, we summarize stress-, aging-, and cancer-associated pH changes together with the mechanisms by which cells regulate cytosolic pH homeostasis. How critical cell components undergo LLPS in response to pH alterations is also discussed, along with the functional roles of intracellular pH fluctuation in the regulation of LLPS. Further studies investigating the interplay of pH with other stressors in LLPS regulation and identifying protein responses to different pH levels will provide an in-depth understanding of the mechanisms underlying pH-driven LLPS in cell adaptation. Moreover, deciphering aging and disease-associated pH changes that influence LLPS condensate formation could lead to a deeper understanding of the functional roles of biomolecular condensates in aging and aging-related diseases.

## Introduction

Liquid–liquid phase separation (LLPS) refers to a demixing transition of an initially homogeneous solution that rearranges and separates into two phases that can coexist stably in solution: a dense and a dilute phase. During this physiochemical process, supersaturated macromolecules are separated from the solution to form a dense phase, while these supersaturated components in the dilute phase are depleted. The macromolecular dense phase has liquid-like properties and can exchange molecules rapidly with the dilute phase [1]. Studies in recent years have shown that LLPS is the driving force for the assembly of nonmembrane organelles and other functional biomolecular condensates, which can achieve spatiotemporal control of their internal complex biochemical reactions without physical barriers [2, 3].

Currently, the formation mechanisms of the resulting condensates formed by LLPS are preliminarily understood and are thought to rely on a network of weak and multivalent protein–protein interactions. Many proteins exhibit LLPS behaviors, and a common feature of such proteins is the presence of multivalent binding domains. Among these, intrinsically disordered regions (IDRs) are the main drivers that provide multivalent interactions [4]. Studies have shown that IDRs are rich in hydrophilic amino acids such as asparagine, glycine, proline, serine, arginine, and aspartate, whereas they are lacking in hydrophobic amino acids such as valine, threonine, leucine, cysteine, isoleucine, histidine, and tryptophan [4, 5]. The enrichment of only a few amino acids in these domains results in low complexity that could mediate weak interactions. Other proteins that contain oligomerization domains and multiple-folded modular domains are also contribute to the multivalent interactions [5]. Furthermore, emerging roles of RNA molecules in the assembly of biomolecular condensates have been revealed. RNAs help establish the promiscuous interaction network through interactions with the RNA-binding domains of proteins, and their intermolecular interactions and self-assembly define the compositions of higher-order condensates [6, 7]. Accumulating evidence shows that protein post-translational modification (PTM) is another important mechanism that achieves cellular control of protein phase separation and condensate formation through processes such as phosphorylation, acetylation, SUMOylation, ubiquitination, methylation, and ADP-ribosylation [8, 9]. These modifications can alter the weak multivalent interactions by changing the charge, structure, hydrophobicity, and other properties of proteins, thus affecting phase separation behavior [8, 9]. In addition, not only protein PTM but also RNA PTM affects condensate dynamics. For instance, N^6^-methyladenosine (m^6^A) of RNA can modulate condensate formation and composition by regulating mRNA distribution into distinct condensates or changing the phase separation behaviors of its binding partners [10, 11].

**Fig. 1:**
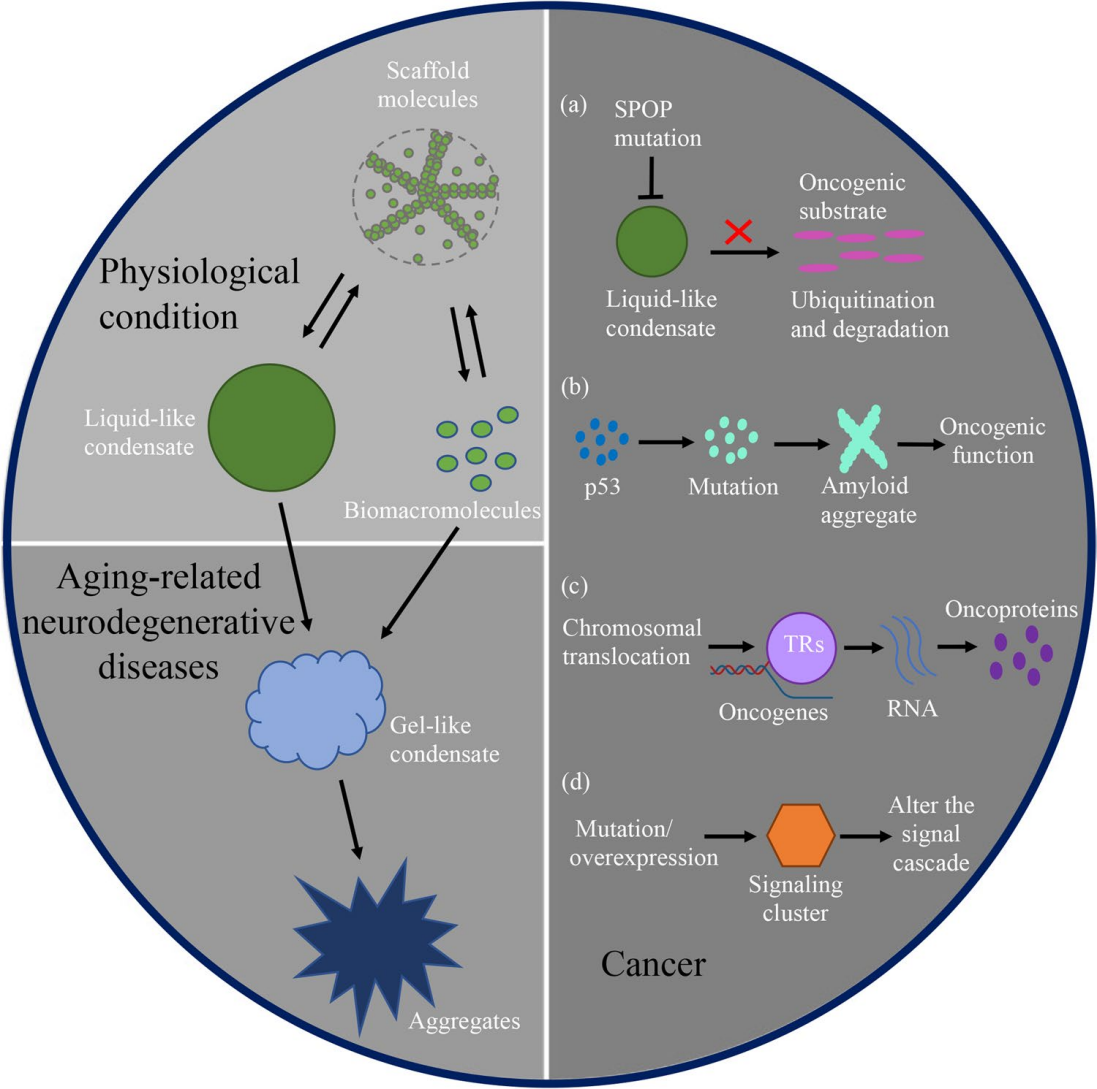
Liquid–liquid phase separation in mammalian cells and its involvement in aging-related neurodegenerative diseases and cancers. Under physiological conditions, scaffold biomacromolecules undergoing liquid–liquid phase separation (LLPS) can interact with and recruit other client molecules to form reversible liquid-like condensates, which participate in a wide range of physiological processes. During aging, multiple factors, including protein mutation and repeated expansions, cellular environmental and metabolic changes, damage to protein quality-control systems, and abnormal protein localization and post-translational modification, can affect the LLPS process and promote aberrant gel-like condensate or pathological protein aggregate formation, ultimately leading to the onset and progression of neurodegenerative diseases. Tumorigenesis is also related to LLPS. **a** Mutations in the substrate recognition domain of the tumor suppressor SPOP prevent its binding to oncogenic substrates and subsequent condensate formation with ubiquitin ligase complex, causing a failure of oncogenic substrate ubiquitination and proteasomal degradation. **b** Mutation of p53 can accelerate its solid-phase transition into amyloid aggregates, which is found in more than 50% of human cancers. **c** Chromosomal translocations lead to aberrant condensate formation of transcriptional regulators (TRs) at enhancers and promoters of oncogenes, driving abnormal oncogenic transcriptional programs. **d** Mutation or overexpression of signaling receptors alter the formation of signaling clusters and activates aberrant signaling cascades, contributing to cancer development

There is mounting evidence that LLPS and condensate formation are widely present in the cells and play important roles in a wide range of physiological processes (Fig. 1), including chromatin organization, cytoskeletal assembly, signal transduction, transcriptional regulation, protein degradation, cell division and differentiation, and environmental response and adaptation [2]. The condensates can transition into different material states such as gel- or solid-like states [1, 12]. Therefore, maintenance of normal condensate material properties can ensure the assembly and disassembly of condensates in a tightly controlled manner to fulfill original functions, while aberrant phase transition is causatively associated with the onset and development of age-related neurodegenerative diseases and cancers (Fig. 1) [13]. In recent research, the development of methods to study LLPS has become an important objective. A series of tools have been developed to predict and analyze the phase separation capabilities of proteins [1]. Fluorescence microscopy observation techniques, including fluorescence recovery after photobleaching (FRAP) and superresolution imaging, can also provide more detailed information on the material properties, composition, and dynamics of biomolecular condensates. In addition, in vitro reconstitution using purified proteins is an accessory method used for studying LLPS [1]. These methods can help researchers to further elucidate the compositions of biological molecules and related biological reactions and explore the factors that drive or influence condensate formation, eventually providing new opportunities for the prevention and treatment of human diseases.

Phase separation of proteins is a multifactor dynamic process, and it occurs not only spontaneously under normal conditions, but also upon stimulation from an array of environmental factors, including changes in temperature, pH, ATP/energy, macromolecule concentration, and ionic strength [14]. These physiological parameters constitute a continuous phase boundary, and crossing this boundary by changing one or more parameters, such as by raising the temperature, depriving nutrients, lowering the pH, or changing other factors, can cause phase separation and the formation of condensates, which is an adaptive tuned response of cells [15]. Homeostasis of pH is a prerequisite for the normal survival of organisms. Many proteins are very sensitive to pH alterations, and a very small change in pH can induce phase transition of proteins. Phase separation in most proteins is triggered at low pH; while in others, it is induced by alkaline pH [16]. In vivo, the mechanism by which pH regulates protein phase separation is not completely clear. Here, we review the literature on stress-associated pH fluctuation in cells, how cells maintain and regulate cytosolic pH, and the effects of pH changes on protein phase separation. The mechanisms by which pH can mediate phase separation are also discussed. Further research on these topics will not only advance our understanding of compartment formation affected by pH changes but will also provide important insight into the relationship between pH and a diverse set of human diseases.

## Diverse stresses induce intracellular pH fluctuation

Cytosolic pH is a tightly controlled physiological parameter in all cellular systems, as almost all cellular processes depend on a constant pH for normal functions [17–21]. For instance, in yeast, pH is involved in replicative senescence of mother cells and rejuvenation of nascent daughter cells [22], and cytoplasmic acidification is critical for yeast cells to enter dormancy under stress conditions [23]. In plants, intracellular pH changes are components of a number of phytohormone signaling pathways, modulating gene expression and defence [21, 24]. In mammals, the maintenance of pH homeostasis is of key importance for the proper execution and regulation of neurotransmission [25]. Small changes in cytosolic pH can lead to major changes in metabolism, signal transduction, protein folding, and protein–lipid interactions [19, 20]. However, organisms are often exposed to diverse adverse conditions throughout their life cycles, and stress-induced cytosolic pH fluctuations are broadly present in the cells; these fluctuations are induced, for example, by osmotic stress, heat shock, and nutrient restriction [26–30]. Aging processes and human diseases, including neurodegenerative diseases and cancers, are also strongly linked with intracellular pH alterations [22, 31, 32]. Table 1 summarizes the stresses that can induce pH fluctuation in the cells of mammals, plants, and microorganisms. Here, we describe the relationships between pH changes and certain stresses, such as temperature perturbation, starvation, and osmotic challenges, as well as aging and aging-related diseases, including neurodegenerative diseases and cancers.

**Table 1:** Effects of stresses on intracellular pH (pHi)

Stress	Species	pHi	Effects of stress	The reason of pH change	References
Heat shock	Yeast	↓	Membrane permeability increase	Protons in the environment influx	[19, 30, 33–35]
	*Drosophila*		Slight cell swelling and altered metabolic activity	Intracellular protons increase	[28, 36, 37]
	Mammals		Inhibit cell growth and change plasma membrane fluidity, permeability to small molecules, and membrane-bound enzyme activity	Inhibition of Na^+^–H^+^ exchange and metabolic pathways	[38–42]
Starvation	Yeast	↓	Decrease cytoplasmic mobility and volume, and cell enters dormancy	Energy shortage to pump protons out	[23, 43–47]
	*Plasmodium*		Block mitosis	Lack of energy to maintain the pH gradients inside and outside the cell	[48]
Osmotic stress	Bacteria	↓	Change cell volume and metabolic processes	Cell loses water and the concentration of protons increases, and activate distinct OmpR-related pathways	[49, 50]
		↑		Proton efflux and osmolarity-stimulated K^+^ uptake	[51–53]
	Protists	↓	Cells shrink, largely rearrange cellular proteins between compartments and decrease activity	Cell loses water and secretes protons	[54–56]
Weak acid	Yeast Bacteria	↓	Decrease cell growth rate and cell growth stasis	Intracellular protons increase and inhibit the ability of cells to maintain normal pH	[45, 46, 57–59]
Hypoxia and anoxia	Mammals Plants	↓	Cytoplasmic acidosis or cell death	Metabolites produced by anaerobic fermentation/ respiration, such as lactic acid	[26, 29, 60–62]
Alcohols	Yeast	↓	Interfere with membrane transport by changing the lipid composition of the plasma membrane	Proton permeability increase	[63, 64]
﻿Pathogen	Plants	↓	Cause pathological damage and activate defense responses	Oxidation burst	[21, 65]
Light intensity	Plants	↑ (light enhance), ↓ (light reduce)	Affect photosynthesis	﻿Proton entering/leaving the ﻿thylakoids	[21, 66]
Aging	Yeast	↑(vacuole)	Cells lose their physical integrity, resulting in impaired function (such as mitochondria/lysosome dysfunction) and increased risk of death or diseases	Pma1 accumulates on the plasma membrane	[22, 67]
	Mammals (Rat hippocampus)	↓		Na^+^–H^+^ exchange may be impaired	[32]
Oxidative stress	*Plasmodium*	↓, ↑ (vacuole)	Lose pH control and decrease intracellular ATP level	Inhibition of V-ATPase activity	[68]
	Mammals	↑ (bovine brain synaptic vesicle)	Reduce neurotransmitter storage and release		[69]
Cancer	–	↑	–	Change the expression and/or activity of plasma membrane ion pumps and transporters that promote H^+^ efflux	[31]

^a^Changes in pHi: increase (↑), decrease (↓)

### Temperature perturbation

Proper environmental temperature is a critical factor for cell survival. When the temperature becomes harsh, organisms must respond rapidly to adapt and thrive. The best-known stress response, the heat shock response (HSR), is a conserved transcriptional program mediated by heat shock factor 1, which is activated upon heat stress. It upregulates the transcription of a set of molecular chaperones to help the cell to manage the accumulation of heat-induced aberrantly folded proteins and aggregates [70]. On the one hand, upregulated chaperones can efficiently refold nonnative proteins or promote the degradation of protein aggregates through autophagy or the ubiquitin–proteasome system (UPS). On the other hand, they can regulate the deposition of certain misfolded proteins into specialized cellular locations to shield them from degradation and to refold them after stress [71]. In addition to HSR, another adaptive mechanism called the unfolded protein response induced by endoplasmic reticulum stress is also activated upon heat exposure and helps to mitigate the damage caused by heat [72, 73]. Moreover, in different research models, ubiquitination-dependent [74–77] and autophagy-dependent degradation [78–82] have been observed to be induced after heat shock, and the activation of these degradation systems is essential for cell survival and recovery from thermally induced protein aggregation [74, 82].

In addition to the activation of evolutionarily conserved systems that contribute to thermotolerance, temperature change is often coupled with fluctuations in cytoplasmic pH [28, 83]. Some studies have shown that heat shock acidifies the cytoplasm. For instance, in yeast, an intracellular pH drop can be induced by heat shock [35]. The same heat-associated pH changes have also been observed in *Drosophila melanogaster* [28] and rat hepatoma cells [41]. Stress-associated acidification is thought to be toxic to cells in some cases [29, 84]; whereas in other cases, it might be a cytoprotective strategy that promotes cellular fitness under stress [23, 33, 85]. For example, cytosolic acidification is required for HSR induction in translationally inhibited cells under heat shock, which allows the cells to adapt to high temperature by increasing the transcription of quality-control components [35, 86]. Furthermore, some stress granule (SG) resident proteins, such as DEAD-box RNA helicase Ded1 [87], poly(A)-binding protein 1 (Pab1) [34], and poly(U)-binding protein 1 (Pub1) [88] in yeast, and Ras-GTPase-activating protein SH3-domain-binding protein (G3BP1) in mammalian cells [89], have been reported to respond to elevated temperatures to undergo LLPS. Likewise, they can also respond to low pH that mimics the pH conditions during heat stress. Therefore, heat-induced acidification may play a key role in protein LLPS following heat exposure and then regulate SG dynamics and cell survival under or after stress.

The mechanism by which heat shock acidifies the cytoplasm is not fully understood. However, evidence has shown that the compositions and structures of the cell membrane are very sensitive to changes in temperature. In yeast, heat shock increases membrane permeability, resulting in proton influx and a rapid decrease in intracellular pH [90]. Studies have shown that intracellular pH disturbance is the triggering mechanism of thermotolerance in yeast [33], and changes in plasma membrane compositions contribute to the thermotolerance of cells, which may also be related to changes in membrane permeability [90]. In turn, heat-induced proton influx and pH decreases can activate plasma membrane ATPase, whose activity is necessary for cell survival under heat shock [33, 91]. The plasma membrane ATPase pumps intracellular protons out of the cell, partially offsetting the internal acidification resulting from the heat-induced increase in membrane permeability [33]. In mammalian cells, heat shock leads to a dramatic loss of plasma membrane Na^+^–K^+^ ATPase activity, which then results in loss of the inwardly directed electrochemical Na^+^ gradient across the membrane [39]. Therefore, it is speculated that Na^+^ gradient-dependent H^+^ export from the cytoplasm to the outside by Na^+^–K^+^ ATPase is affected and that the cytoplasm is acidified during heat stress.

### Starvation

A decrease in cytosolic pH can also be caused by starvation. In yeast cells, numerous studies have shown that the cytoplasmic pH decreases from approximately 7.4 to approximately 6.0 under starvation conditions [43, 45, 47]. Pma1, the plasma membrane-localized P-type H^+^-ATPase in yeast, is involved in pumping protons out of the cells and is a primary contributor to the maintenance of cytosolic pH stability near neutrality [92, 93]. Importantly, its activation requires glucose-regulated phosphorylation [94]. In addition, other pumps, such as V-type H^+^-ATPases (V-ATPases), are also responsible for cytosolic pH regulation [95]. They work by pumping excess protons into the vacuole to regulate cytosolic pH homeostasis; they also maintain effective localization of Pma1 at the plasma membrane [95, 96]. Glucose is also required for V-ATPase activation because it mediates reversible associations between the V1 and V0 domains of V-ATPase [43, 97]. Under favorable conditions (with glucose), V-ATPase cooperates with Pma1 to pump protons out of the cytoplasm and help cells stabilize cytoplasmic pH in an ATP-dependent manner. However, upon glucose depletion, a drop in cytoplasmic pH is observed, as starved yeast cells lack efficient H^+^-ATPase assembly and activation to support the proton gradient across the membrane [46]. Intracellular protons cannot be discharged outside of the cell. Instead, they accumulate inside the cell; thus, cytosolic pH decreases. The increased concentrations of intracellular protons cause the phase transition of the cytoplasm from a fluid-like to a solid-like state, and such a dormant or quiescent state is a protective strategy for cell survival under conditions of starvation [23]. Likewise, evidence suggests that nutrient supply is also closely related to cytoplasmic pH in *Physarum plasmodium*. The cycle of intracellular pH corresponds to the period of the cell cycle of *P. plasmodium*. When *P. plasmodium* is growing in non-nutrient medium, the intracellular pH remains stable and then begins to decline gradually, which serves to block normal mitosis. However, upon refeeding of starved *P. plasmodium* with the nutrient medium, intracellular pH can recover to normal values and the cell cycle resumes [48].

### Osmotic stress

In addition to heat shock and starvation, osmotic stress is another important environmental factor affecting cell survival and growth. Organisms including microbes, plants, and mammals, are commonly confronted with hyperosmotic conditions, which trigger a series of actions resulting in downregulation of cellular activity and progression of disease [98–100]. When osmolarity changes, cells adjust their volumes accordingly in response to the changing environment. Cells mainly regulate volume changes by controlling substance influx and efflux, which is usually manifests as cell contraction or expansion, so that cells can return to a normal resting state [101–103]. A variety of membrane transporters are involved in this complex regulation process. For example, in a hypotonic environment, mammalian cells initially expand via water uptake and subsequently undergo compensatory shrinkage to partially regulate volume reduction, usually through efflux of KCl and organic osmolytes [104, 105]. In contrast, in hypertonic environments, cells undergo transient dehydrating contraction by absorbing Na^+^, K^+^ and CI^−^ and then pumping out Na^+^ to regulate the increase in cell volume [104].

Intracellular osmotic homeostasis is necessary to maintain normal cell function and survival, and osmotic dysregulation is the basis of many diseases and their complications, including cataracts [106], epilepsy [107], inflammation [100, 108], and hypernatremia [109]. For instance, in hyperglycemia or hypergalactosemia, activated aldose reductase converts glucose and lactose to galactose and sorbitol, respectively, which accumulate in the lens and cause osmotic swelling, leading to diabetic cataracts [106]. In addition, cancer and aging processes are also closely related to intracellular osmotic regulation. Many studies have shown that ion channels and ion pumps are beneficial to the development and progression of cancer [110]. Given the importance of ion channels for osmotic homeostasis and the abnormal expression of transporters in many cancers [111], it is likely that the original homeostasis in cells will be disrupted, creating a more favorable internal environment for cancer development. Interestingly, Yes-associated protein (YAP), is a transcriptional coactivator that is widely activated in cancer cells [112], can sense the tumor microenvironment and modify the physicochemical properties of the surrounding environment by activating transcription, thereby promoting tumor development [113]. Moreover, YAP-activated transcription is mediated by the LLPS process, which also occurs under hypertonic conditions [114]. Aging does not directly cause disease, but in this process, the homeostasis of water in the human body is often disturbed [115]. Thus, normal osmotic regulation is impaired, and this impairment is followed by increases in the incidence and severity of diseases, such as hypoosmolality and hyperosmolality [116].

Interestingly, a growing body of evidence implicates hyperosmotic stress as a factor leading to internal pH alteration. In *Listeria monocytogenes*, a ubiquitous gram-positive food-borne pathogen, the initial response to osmotic stress caused by sorbitol or NaCl is a decrease in intracellular pH [50]. Hyperosmotic stress also leads to cytosolic acidification in *Dictyostelium discoideum*, which works as a novel signal mediator responsible for hyperosmotic stress responses [54]. Moreover, another study has indicated that hyperosmotic shock elicits a transient increase in *Escherichia coli* cytoplasmic pH, but the pH returns to normal values after osmotic adaptation [52]. However, whether and how osmotic dysregulation in mammalian cells alters pH and whether it is relevant to human diseases remain unclear; thus, these aspects require further investigation to advance our understanding of pH-related condensate formation and diseases.

Taken together, the evidence indicates that diverse environmental alterations contribute to intracellular pH fluctuation. Manipulating intracellular pH not only serves to maintain the morphology and function of cells to ensure normal growth and metabolic activities, but also is associated with the preservation of cellular equilibrium in response to several environmental factors, which could promote cellular fitness.

### Aging and neurodegenerative diseases

Aging is usually an irreversible biological process and is considered to be a predominant risk factor for many neurodegenerative diseases [117]. Nine hallmarks of aging have been tentatively identified in different organisms, including genomic instability, telomere attrition, epigenetic alterations, loss of proteostasis, deregulated nutrient sensing, mitochondrial dysfunction, cellular senescence, stem cell exhaustion, and altered intercellular communication. These hallmarks can be classified into three layers: primary hallmarks, antagonistic hallmarks, and integrative hallmarks, which co-occur during aging and are usually interconnected with each other; defining the exact relationships and causal network of these hallmarks may contribute to future studies on aging and aging-related diseases [118].

**Fig. 2:**
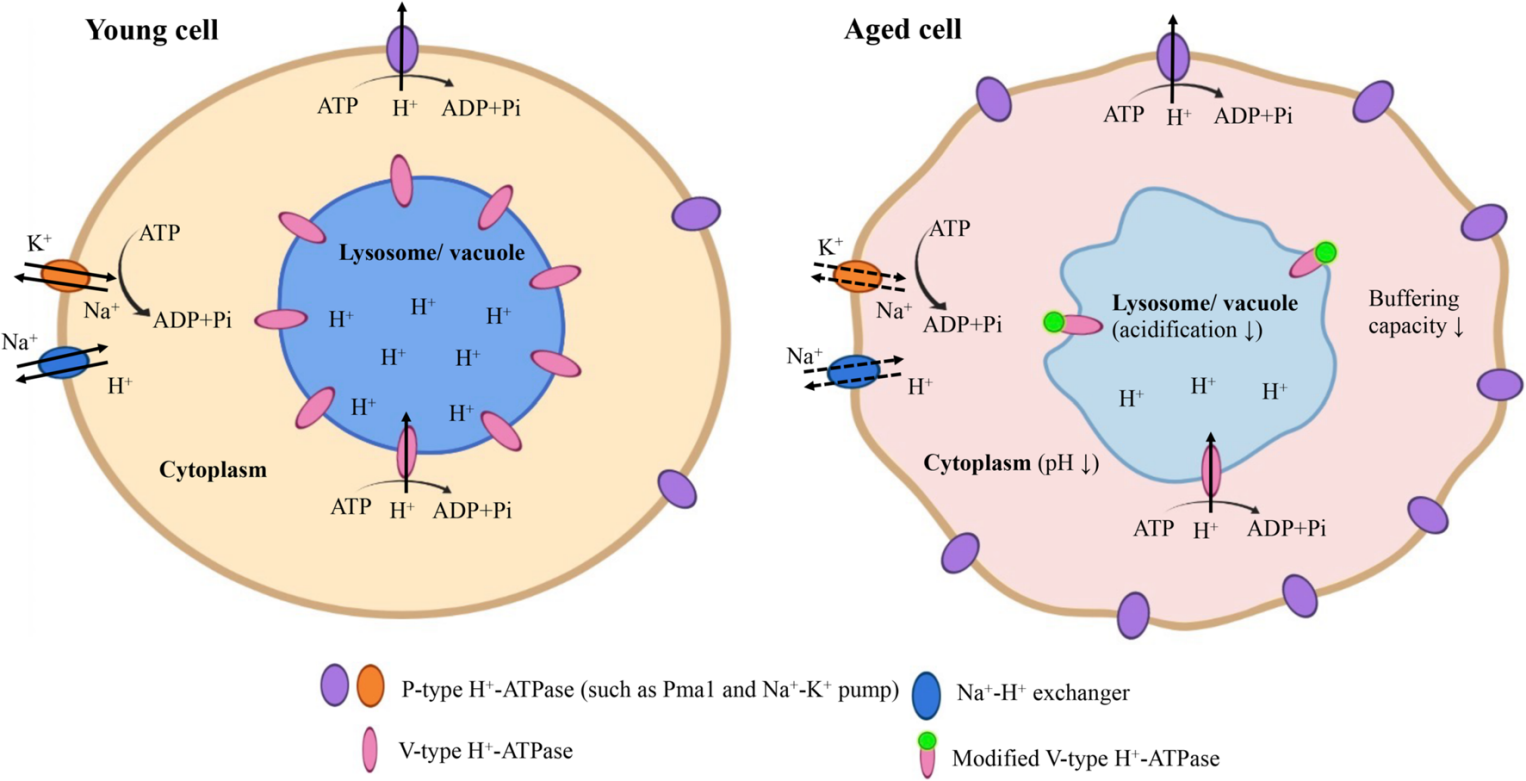
Aging affects intracellular pH. When cells are young, P-type H^+^-ATPases distributed on the plasma membrane act in concert with V-type H^+^-ATPases localized on the lysosomal/vacuolar membrane to regulate intracellular pH. However, during aging, for example, in yeast, P-type H^+^-ATPase Pma1 accumulates on the plasma membrane, and excessive H^+^ is pumped out of the cell, resulting in reduced cytosolic H^+^ availability for V-type H^+^-ATPase. This leads to a decrease in vacuolar acidity. In other cases, such as in the aged rat hippocampus, the Na^+^–K^+^ pump and Na^+^–H^+^ exchange may be impaired; as a result, H^+^ accumulates in the cytoplasm, and cytosolic pH decreases. Moreover, cell buffering capacity is also impaired during aging. V-type H^+^-ATPase is a target of oxidative stress in aging. Increased oxidative modification of V-type H^+^-ATPase might inhibit V-type H^+^-ATPase-mediated vacuolar acidification. Alternatively, aging might alter lysosomal/vacuolar acidification by downregulating V-type H^+^-ATPase subunit expression, lowering the availability of V-type H^+^-ATPase. The solid lines represent normal ion transport. The dashed lines represent impaired ion transport. In the young cell cytoplasm, yellow represents cytoplasm with a normal pH. In the aged cell cytoplasm, red represents cytoplasm with a decreased pH

In addition to the hallmarks of aging discussed above, growing evidence shows that intracellular pH alterations are also intimately linked to aging processes and aging-related neurodegenerative diseases. In mammals, the intracellular pH of central neurons is tightly regulated, and its fluctuations are important for signaling and synaptic plasticity [119, 120]. Specifically, in cortical neurons, a mild intracellular pH decrease occurs following an excitability increase, and this decrease acts as feedback to reduce local bioelectric activity and excitability. However, when the intracellular pH is outside a certain range and reaches its limits, there may be an increased risk of cell death [25, 119, 121, 122]. Importantly, a decrease in neural pH levels has been observed in a number of neurodegenerative disorders [123, 124] and even in the normal aging process [32, 125, 126]. Moreover, acute neuroinflammation has been observed to provoke intracellular acidification in the mouse hippocampus [127]. For example, in mammalian cortical neurons, intracellular pH is negatively correlated with aging, as evidenced by significantly lower pH in hippocampal slices from aged rats than in slices from young rats [32, 128]. Likewise, in human neurons, the intracellular pH has also been observed to decrease with aging [126, 129]. The mechanisms involved in decreased intracellular pH may be the disruption and overwhelmed of pH regulatory systems through processes including aging-related decreases in buffering capacity and disruption of diverse transmembrane acid/base-transporters [32, 128–130]. For instance, considering that Na^+^–H^+^ exchange is the dominant regulatory mechanism for proton extrusion in cultured hippocampal neurons, altered H^+^ homeostasis might be attributable to impaired Na^+^–H^+^ exchange (Fig. 2), which utilizes the inwardly directed electrochemical Na^+^ gradient generated by Na^+^–K^+^ ATPase to export H^+^ [32]. Moreover, limited ATP synthesis during aging might also affect ATP-driven ion pumping, including Na^+^ gradient generation by Na^+^–K^+^ ATPase [131]. The impacts of aging-related alterations on pH regulation are controversial. A slight decrease in intracellular pH may provide neuroprotection [132], while successively greater acidification may increase the vulnerability of brain tissue to stressful conditions [125, 133–135].

In addition to cytosolic pH dysregulation, lysosomal/vacuolar pH dysregulation has also been implicated in aging and aging-related neurodegenerative diseases. Evidence is now emerging that defective lysosomal function is a major factor in the pathogeneses of different types of neurodegenerative diseases, specifically, a failure of the maintenance of a highly acidic lysosomal/vacuolar pH [136]. There is also increasing evidence for aging-related compromise of lysosomal function [22]. In yeast, vacuolar pH is a critical regulator of mitochondrial function and replicative lifespan. Vacuolar acidity declines with aging, and reduced vacuolar acidity disrupts pH-dependent amino acid homeostasis in the vacuolar lumen, resulting in age-related dysfunction of mitochondria and a shortened lifespan [67]. In addition, lifespan extension via calorie restriction and methionine restriction requires vacuolar acidification [67, 137, 138]. The decrease in vacuolar acidification in yeast is due to excess accumulation of the major regulator of cytosolic pH, Pma1, in mother cells (Fig. 2). Vacuole acidity is thus antagonized by reduced cytosolic proton availability [22]. Importantly, V-ATPase is implicated in lysosomal acidification. Mutations in V-ATPase or proteins that regulate V-ATPase function are observed in aging-related neurodegeneration [136]. It is conceivable that during aging, oxidative stress might impair V-ATPase activity through increased oxidative modification of V-ATPase (Fig. 2), which is inspired by the observation that hydrogen peroxide inhibits bovine brain synaptic vesicle V-ATPase activity [69]. In fact, increased oxidative modification of V-ATPase subunits has been observed in aged rat brain tissue [139], and oxidative modification is known to impair the activity of certain enzymes [140, 141]. Alternatively, aging might alter lysosomal/vacuolar acidification via dynamic transcriptional regulation of V-ATPase subunits (Fig. 2), a mechanism that is supported by the observation of reduced V-ATPase subunit mRNA levels in hippocampal neurons in sporadic Alzheimer’s disease (AD) [142]. In conclusion, intracellular pH alterations, including cytosolic pH changes and lysosomal/vacuolar pH dysregulation, are also striking features that occur during the aging process and aging-related diseases onset.

The processes of aging and aging-related neurodegenerative diseases onset are typically accompanied by the formation of widespread intracellular protein aggregates [143]. Many RNA-binding proteins, such as fused in sarcoma (FUS), tau, alpha synuclein (α-Syn), and TAR DNA-binding protein 43 (TDP-43), are the main components of protein inclusions or aggregates in diverse neurodegenerative diseases, including amyotrophic lateral sclerosis (ALS) [144], frontotemporal dementia (FTD) [144, 145], Parkinson’s disease (PD)[146], and AD [147]. Furthermore, these disease-associated proteins are well known to undergo LLPS, and the failure to maintain their liquid-phase homeostasis may serve as a trigger of solid protein aggregate formation (Fig. 1) [148]. Diverse layers of regulation may affect their transition from a liquid-like state with physiological function to solid pathological aggregates. Therefore, it is reasonable to speculate that alterations in the intracellular microenvironment, such as pH changes during aging, provide these phase-separated neurological disorder-related proteins with the opportunity to change their phase separation behaviors and increase the risk of aggregation. In fact, evidence has already shown that LLPS of α-Syn and its subsequent maturation into protein aggregation are pH-mediated [149]. Therefore, further investigations on aging-induced pH dysregulation will not only advance our understanding of aberrant LLPS and compartment formation but will also provide important insight into the onset of aging-associated pathologies.

### Cancers

In recent years, increasing evidence has linked LLPS and condensates to tumorigenesis. A growing number of cancer-associated proteins have been reported to have the ability to undergo LLPS and form biomolecular condensates, such as speckle-type POZ protein, which is involved in oncogenic substrate degradation [150]; p53-binding protein 1 (53BP1) and FET proteins, which are involved in the DNA damage response and genomic stability [151, 152]; and EWS-FLI1, β-catenin, YAP, and PDZ-binding motif (TAZ), which are involved in transcriptional regulation [114, 153–155]. In all of the above cases, disrupting functional condensate assembly of tumor suppressors or promoting aberrant condensate assembly of oncoproteins contributes to the oncogenic process (Fig. 1a–d). In addition, aberrant assembly of other membrane-less compartments formed by LLPS, including SGs [156, 157], PML bodies [158], paraspeckles [159], and amyloid bodies [160], is also associated with cancer. The tumor suppressor p53 has been the “star molecule” of molecular biology and oncology since its discovery. It acts as a transcription factor, activating or inhibiting the transcription of various downstream target genes involved in cell cycle regulation, senescence, and apoptosis [161, 162]. p53 prevents tumor development through cell cycle arrest, DNA repair, and antioxidant protein production to maintain genome integrity and limit cell proliferation under adverse conditions such as DNA damage, hypoxia, oncogene expression, nutrient deprivation, and ribosomal dysfunction [162–164]. Moreover, its mutation, which tends to result in protein aggregation, is found in more than 50% of human cancers [165, 166]. Recent evidence has revealed that the p53 core domain can undergo LLPS and then undergo a phase transition to the solid-like state. Mutation of p53 can accelerate its solid-phase transition into amyloid aggregates (Fig. 1b) [167]. Therefore, it is a reasonable assumption that differences in the tumor microenvironment compared to the microenvironment of normal differentiated cells may trigger certain proteins to undergo LLPS and phase transition to solid aggregates, leading to further cancer progression.

As cancer cells grow at an uncontrolled high rate, they are usually challenged with an adverse macroenvironment characterized by hypoxia and nutrient starvation [168]. Apart from this, considerable evidence links cancer directly to pH alterations since a higher intracellular pH and a lower extracellular pH than those of normal differentiated cells are observed in most cancers, regardless of tissue origin and cell type [169, 170]. These differences may be attributable to changes in the expression and/or activity of plasma membrane ion pumps and transporters, as well as changes in metabolic activities [169, 170]. In turn, the increased intracellular pH and the decreased extracellular pH also synergistically enhance cancer progression. On the one hand, the increased intracellular pH can increase cell proliferation, facilitate apoptosis evasion, and promote cytoskeletal remodeling for cell migration. On the other hand, the acidified extracellular environment can increase the activities of acid-activated proteases and promote extracellular matrix degradation, thereby accelerating tumor cell invasion and dissemination [31]. However, during these processes, whether and how pH alterations of cancer cells are related to the aberrant phase behavior of cancer-related proteins or aberrant formation of membrane-less compartments such as SGs, PML bodies, paraspeckles, and amyloid bodies, remains unclear and requires further in-depth investigation. Such research will provide more knowledge about the molecular basis of cancer and facilitate the development of new therapies.

## Cytosolic pH control by metabolism-based and transporter-based regulation

Since pH control is a critical requirement for growth in all organisms, different organisms have adopted a number of common strategies to address the challenges of pH maintenance in the face of rapid metabolism and extracellular environment changes [171–173]. Cells separate metabolites, proteins, and biochemical processes in a manner dependent on compartmentalized membrane-bound organelles, each of which has distinct pH requirements and pH regulation mechanisms [172]. More importantly, cellular compartments have inherent pH buffering capacities. This buffering is achieved by the presence of various intracellular weak acids and bases, as well as the ionizable groups of macromolecules such as side chains of amino acids [174]. Moreover, cytosolic pH regulation also relies on metabolites produced by pH-dependent biological reactions [175]. Organic acids such as malate can produce or consume H^+^ via carboxylation and decarboxylation reactions. Therefore, correct synthesis, degradation, and transport of organic acids through the cytoplasm to other organelles are thought to be important strategies to regulate intracellular pH [176, 177]. In addition, the alternative pathways to glycolysis, the cyanide-resistant alternative respiration pathway and malate-derived lactic and alcoholic fermentation, which are unique to plants, jointly regulate pH homeostasis in plants [175]. In mammalian cells and fermenting yeast, CO_2_ produced during metabolism can diffuse freely through biological membranes. It can react with water to form HCO_3_^−^, which is an effective proton buffer and consumes protons to produce carbonic acid when the cells are confronted with an acute drop in intracellular pH [178, 179].

However, when cells are under long-term stress, the major regulatory mechanism to maintain cytosolic pH homeostasis is the membrane transport of H^+^, which involves a large array of distinct transport pathways. For example, P-type proton pumps are widely distributed on eukaryotic cell membranes, and they are the main determinants of proton efflux and cytoplasmic pH control in plants and yeast [19, 173]. As mentioned above, Pma1 is the most abundant protein in the plasma membrane of yeast and actively coordinates with V-ATPases to regulate cytosolic pH [19, 173]. V-ATPases can also acidify compartments in an ATP-dependent manner and are distributed in acidic organelles such as the Golgi apparatus, vacuole/lysosomes, and endosomes of all eukaryotic cells [180]. In yeast cells, V-ATPase activity is indispensable for vacuolar acidification during glucose metabolism and homeostasis of cytoplasmic pH in the short term. In the long term, V-ATPase is very important for the stability of Pma1 localization [95]. F-type proton pumps are mainly distributed in the bacterial plasma membrane, the mitochondrial membrane, and the plant endomembrane. In enterococci, when the cytoplasm is acidified, the level and activity of F-type H^+^-ATPase increase synchronously, leading to cytoplasmic alkalization [181]. When the pH value is restored to the initial value, the decrease in the amount and activity of the enzymes terminates proton extrusion. Thus, changes in the amount and activity of enzymes seem to be necessary for pH regulation [182].

Moreover, these proton pumps act in concert with a large array of other transporters. Increasing evidence indicates that a number of ion/H^+^ exchangers are also important for intracellular pH regulation in different organisms, including yeast, plants, and mammals [19, 172, 173]. These exchangers couple the transfer of H^+^ across biological membranes to counter-transport of other cations, such as Na^+^ or K^+^, to protect against excess acidification. Furthermore, Na^+^-coupled HCO_3_^−^ transporters, which are involved in the uptake of extracellular HCO_3_^−^, have also been reported to play key roles in the regulation of cytosolic pH. They contribute to the maintenance of CO_2_–HCO_3_^−^ equilibrium, the most important pH buffering system [183, 184]. Although the importance of proton extrusion in pH control has been revealed, acid-importing transporters such as Cl^−^–HCO_3_^−^ exchangers, which allow HCO_3_^−^ efflux, can efficiently prevent overalkalization of the cells by working counter to CO_2_–HCO_3_^−^ transporters to enable the fine control of cytosolic pH [185].

In summary, cells exhibit a complicated pH regulation network dependent on the interplay among multiple transporters that import or export proton equivalents and metabolism-based regulatory mechanisms, and this network can accurately regulate and maintain cytosolic pH. More details can be found in recent reviews [19, 171–173].

## pH-dependent phase separation condensate formation induced by stress

As discussed above, many types of stress cause a decrease in cytoplasmic pH, and these stress conditions are known to induce phase separation of proteins to form condensates. Here, we summarize the proteins that are known to form phase separation condensates in response to pH stress together with other stresses in which phase separations are mainly affected by pH alterations, such as heat shock and starvation (Table 2).

**Table 2:** Proteins undergoing LLPS and response to stress-induced pH changes

Protein	Function	Domain function in LLPS	RNA^a^	Species	Stress	Effects of pH	References
Ded1p	Initiate translation	IDR	+	Yeast	Acidic pH, heat shock	−	[87, 186–188]
Pab1	Control mRNA polyadenylation, stability, and translation	LCR, RRMs	−	Yeast	Acidic pH, heat shock	Act as signal messenger and affect electrostatic interaction	[34, 189, 190]
Pub1	Regulate translation	LCR, RRMs	−	Yeast	Acidic pH, heat shock, glucose starvation	Affect protein solubility and electrostatic interaction	[88, 191]
DDXs	Coordinate mRNA de-capping and decay, regulate general translational repression	LCDs	+ , − (if excess)	Bacteria, Yeast, Mammals	Acidic pH, glucose starvation	−	[192–194]
Gln1	Promote the conversion of glutamate into glutamine	−	−	Bacteria, Yeast	Acidic pH, glucose starvation	Act as signal messenger	[47, 195–197]
Sup35	Terminate translation	The N-terminal prion domain and the electrically neutral domain	−	Yeast	Acidic pH, glucose starvation	Act as signal messenger	[44, 198]
Atg1 complex	Participate in PAS assembly	IDRs	−	Yeast	Acidic pH, glucose starvation	Possible act as signal messenger	[199–202]
G3BP1	Promote SG assembly and inhibit RNA aggregation	IDRs, nuclear transport factor like domain, RBD	+	Mammals	Acidic pH, heat shock, osmotic stress	Affect protein solubility and electrostatic interaction	[89, 203]
SARS-CoV-2 N protein	Participate in viral RNA replication and virion packaging	IDR1	+	Virus	Acidic pH	Affect electrostatic interaction	[204–206]
α-Syn	Act as a SNARE-complex chaperone and contribute to Parkinson’s disease pathogenesis	The N-terminal region (most family disease mutations occur) and the “non-amyloid-β component” region	−	Mammals	Acidic pH	Affect electrostatic interaction	[149, 207–209]
4R-Tau	Induce tubulin assembly and stabilize microtubules	The microtubule-binding repeats	−	Mammals	Lower-critical solution transition	Affect electrostatic interaction	[16, 210–212]
FUS	Participate in DNA repair, transcription, and RNA biogenesis	LCDs	+ , − (high ratios)	Mammals	Acidic pH, DNA damage, heat shock	−	[213, 214]
53BP1	Regulate the DNA damage response and p53 signaling	The oligomerization domain and BRCT domain	−	Mammals	Acidic pH, DNA damage, light	−	[151, 215, 216]
ELPs	New biomaterials for drug delivery and tissue engineering	−	−	Artificial synthesized	Lower-critical solution transition	Possible affect protein solubility and electrostatic interaction	[217–221]

^a^Effects of RNA on phase separation: promoting/requiring ( +), inhibiting (−)

Many biomolecules undergo LLPS to form liquid-like condensates that mediate diverse cellular functions [222, 223]. For example, autophagosome formation is a process that is precisely regulated by protein phase separation. Atg1 complex formation is a prerequisite for preautophagosomal structure (PAS) assembly and autophagy initiation [202]. Recent research suggests that the PAS is a liquid-like condensate formed by phase separation of the Atg1 complex, which is critical for further dynamic recruitment of other proteins or factors during autophagosome formation. Notably, this process occurs under low pH and starvation conditions [200–202]. TORC1 is a modulator of PAS organization that targets Atg1 complex assembly by regulating the phosphorylation/dephosphorylation of Atg13, a component of the Atg1 complex [202, 224]. Its activity is also modulated by phase-separated compartments such as SGs. Under stressful conditions, including heat, starvation, and osmotic stress, TORC1 is recruited into SGs; as a result, TORC1 signaling is inhibited [225–227]. For example, in yeast, TORC1 is partitioned into heat shock-induced SGs, which then prevents an increase in the frequency of heat-induced DNA mutations [225]. Under osmotic stress, TORC1 in mammalian cells is similarly sequestered into SGs, thereby blocking its signal transduction to downstream effectors [227].

SGs are also dynamic membrane-less organelles, the formation of which is driven by LLPS [228, 229]. It has been reported that many proteins in SGs exhibit phase separation behavior under stress-associated pH changes. Pab1, is an RNA-binding protein consisting of a short N-terminal sequence, four RNA recognition motifs (RRMs), a proline-rich low-complexity region (LCR) and a C-terminal peptide-binding domain. It plays a key role in controlling the polyadenylation, stability, and translation of mRNA in yeast cells [34, 190]. Pub1 is similar to Pab1 in that it is an RNA-binding protein with three RRMs and one LCR [191]. Both Pub1 and Pab1 are core components of SGs and are prone to phase separation when temperature increases, pH decreases, or nutrients are lacking to help cells survive during stress [34, 88, 189]. In addition, G3BP1 is a central node and molecular switch in SG assembly. Its phase separation also occurs in an RNA-dependent manner under low pH and heat shock [89, 203]. Moreover, members of the Asp–Glu–Ala–Asp (DEAD)-box ATPase (DDX)^3^ family are widely present in both eukaryotes and prokaryotes [192, 194], and studies have suggested that many proteins in the DDX family undergo LLPS in vivo or in vitro, including Ded1, Dbp1, and Dbp2 in yeast; DDX3X, DDX4, and DDX6 in humans; and DeaD, SrmB, and RhlE in *E. coli* [192, 193]. Ded1p, an ATP-dependent DEAD-box RNA helicase in yeast, is an indispensable translation initiation factor and a component of SGs [188]. It can parse the secondary structure of mRNA 5′ untranslated regions for ribosomal scanning and recognition of the initiation codon [186, 187]. Studies have shown that Ded1p undergoes phase separation and forms condensates at elevated temperatures, or at lower temperatures when the pH is adjusted to that of the heat-stressed cytosol (heat-shocked cells experience a decrease in cytosolic pH). When in condensate form, Ded1p is translationally inactivated, which leads to a switch in translation from housekeeping transcripts to stress-responsive transcripts [87]. Therefore, heat shock-induced and temperature-associated pH change-induced Ded1p condensation in SGs is an adaptive response to survive heat shock. It promotes an evolutionarily conserved heat shock response that selectively translates housekeeping or heat shock transcripts [87]. Similarly, another DDX family member in yeast, Dhh1, is responsible for the assembly and disassembly of RNA-containing membrane-less organelles. Dhh1 also exhibits enhanced phase separation at low pH, which mimics the pH conditions during glucose starvation [192].

Moreover, evidence indicates that in changed growth conditions, enzyme activities can be acutely regulated through the formation of phase separation-induced enzyme condensates, which restrict or promote specific biochemical reactions in membrane-less organelles, suggesting the importance of phase separation in regulating the metabolism of cells [47, 195]. For instance, glutamine synthetase (Gln1) is an indispensable metabolic enzyme that catalyzes the synthesis of glutamate and ammonium into glutamine, a process that requires ATP. Gln1 forms filaments during a state of advanced cellular starvation, and filament formation leads to enzymatic inactivation [197]. Further evidence demonstrates that starvation-induced cytosolic acidification is the trigger for Gln1 condensate formation, and many metabolic enzymes follow this principle to help cells endure and recover from severe starvation conditions [47].

In addition to the above-mentioned findings, there are other proteins for which LLPS is directly or indirectly affected by pH changes, increasing cell fitness or inducing diseases. For instance, Sup35 is a translation termination factor in budding yeast [198]. It can form condensates upon energy depletion or at a low pH. This pH-dependent phase separation of Sup35 can serve as a means for Sup35 to rescue itself from stress-induced damage and promote recovery of the yeast cell from stress [44]. The nucleocapsid protein (N) of the severe acute respiratory syndrome coronavirus (SARS-CoV-2) is a multivalent RNA-binding protein that is essential for viral RNA replication and virion packaging [206]. The N protein can partition into SGs and interact with G3BP1/2 to block the assembly of SGs through RNA-dependent liquid phase separation and thus disrupt the immune response of host cells [204]. Notably, phase separation of the N protein occurs under physiological conditions and is enhanced at low pH [205]. α-Syn is an IDP for which aggregation into amyloid-like fibrils is associated with PD pathology [207, 208]. One study found that α-Syn initially undergoes phase separation and becomes rigid over time and eventually transforms into solid-like aggregates. Low pH can promote α-Syn LLPS and further increase the maturation and nucleation of α-Syn aggregates, which is relevant to PD pathogenesis [149]. Additionally, pathological inclusions of the microtubule-associated protein Tau have been reported to accumulate in patients with several neurodegenerative diseases [210–212]. Evidence indicates that the microtubule-binding repeats of the Tau protein have a strong propensity for liquid demixing, which occurs over a wide range of pH values. The phase separation of these four repeats at different pH values wound concentrate the most aggregation-prone Tau residues and further promote amyloid formation [16]. Interestingly, in addition to natural proteins, artificially constructed polypeptides can also undergo phase separation. Elastin-like polypeptides (ELPs) are recombinant protein polymers composed of pentapeptide (Val–Pro–Gly–Xaa–Gly)_*L*_ repeat units, which are recurring motifs in tropoelastin in a wide range of species. ELPs are often used as new biomaterials for drug delivery and tissue engineering [218–221]. One study found that ELPs can exhibit reversible phase separation triggered by a wide range of pH values, and this pH responsiveness is controlled by the type and number of ionizable residues and the molecular weight of the ELPs. This property of specific pH-controlled ELP phase separation can be applied in drug delivery systems for local cancer therapy, as various tumors types usually have different pH values than healthy tissues [217].

Finally, as we discussed above, many cancer-associated proteins have been reported to undergo LLPS and to be involved in biomolecular condensate formation. 53BP1 is a binding partner of p53 [230] that can directly regulate p53 and affect p53 target gene expression [231]. It is also one of the main regulators of the DNA damage response, loss of which has been associated with apoptosis and cancer cell proliferation [232]. Studies have found that 53BP1 undergoes LLPS at DNA damage sites, forming DNA repair condensates that recruit and stabilize p53 [151]. If the expression of 53BP1 is changed or LLPS behavior is affected, the disruption of condensate formation leads to destabilization of p53 and reduced induction of its target genes as well as cell cycle arrest [151]. Interestingly, it has been reported that 53BP1 can respond to low pH to form 53BP1 droplets [151]; thus, further studies on the relationships of pH regulation and 53BP1 LLPS will help enhance our understanding of tumorigenesis. However, besides 53BP1, research on the relationships between cancer-associated proteins and pH dysregulation are limited. Considering that the physiochemical properties and microenvironment of cancer cells are different from those of normal cells [168], two important research topics in the future are whether these proteins undergo pH-regulated LLPS and how pH-regulated LLPS is relevant to tumorigenesis. In addition, research on how microenvironmental changes in cancer cells affect the dynamics of intracellular membrane-less organelles such as SGs, PML bodies, paraspeckles, and amyloid bodies, whose aberrant assembly is associated with cancer, is also needed. Such research will provide further evidence regarding the links among pH, LLPS, and cancer.

## Mechanisms underlying pH-mediated phase separation

### pH changes mediate protein–protein/RNA interactions

**Fig. 3:**
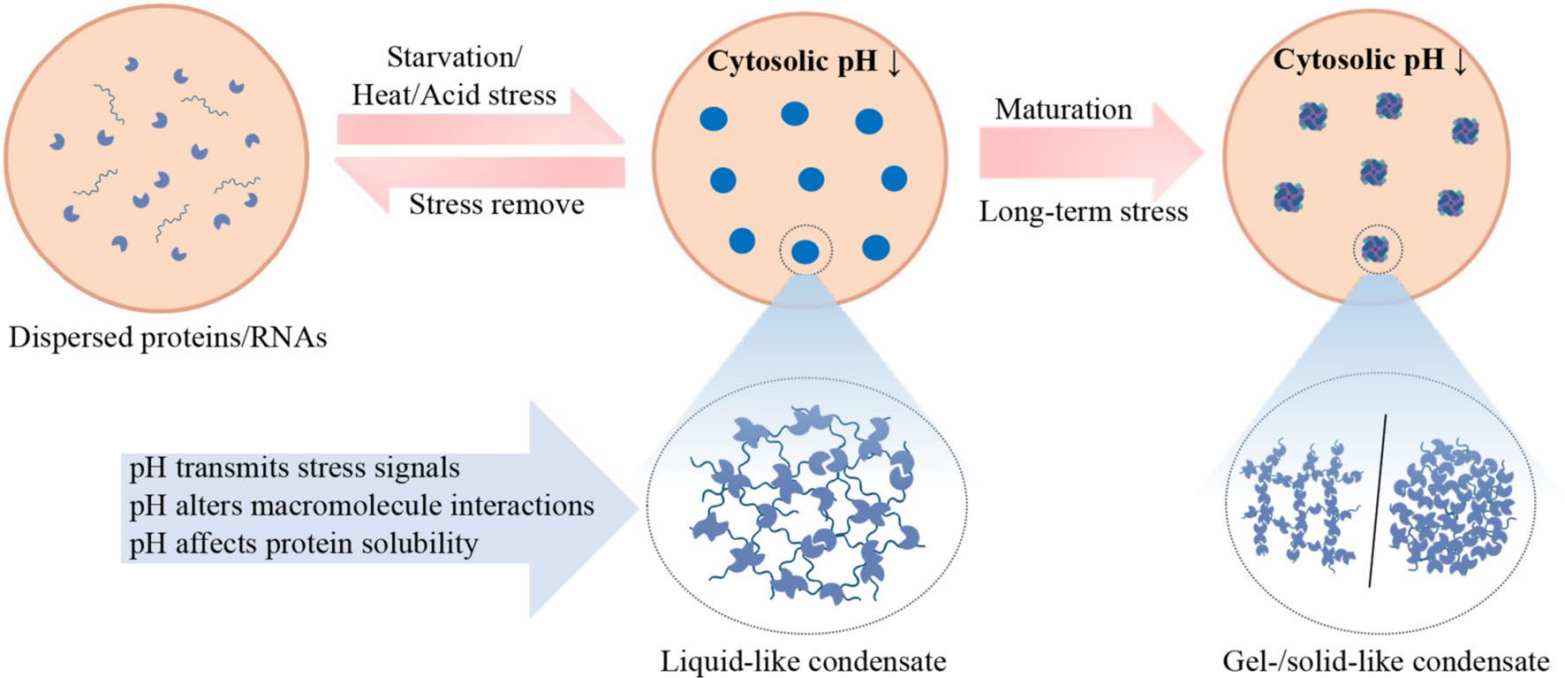
Roles of pH in biomolecular condensate formation. Under nonstress conditions, proteins and RNAs are dispersed in the cytoplasm. When cells are exposed to stresses such as starvation, heat shock, or acid stress, the intracellular pH changes, and this change is accompanied by the formation of protein- and RNA-containing biomolecular liquid-like condensates. During this process, pH plays multiple functional roles in triggering liquid–liquid phase separation (LLPS)-driven condensate formation; for example, it affects protein–protein/RNA interactions, alters protein solubility, or acts as a messenger to transmit stress signals. pH changes can also enhance phase separation, which may gradually mature and result in transformation into an irreversible gel-/solid-like state

Some proteins or their specific domains possess the ability to sense stresses directly and thus undergo phase separation in response to these stresses [34, 88]. It is known that LLPS is driven by multivalent weak macromolecular interactions (protein–protein, protein–RNA, and RNA–RNA interactions), disruption or alteration of which would affect protein phase separation behaviors [5, 233]. Therefore, pH changes can influence intramolecular or intermolecular protein–protein/RNA interactions by changing the net charges of components, thereby driving LLPS (Fig. 3). For example, G3BP1 is a multidomain protein composed of two folded domains and two IDRs. Under nonstress conditions, its central negatively charged, glutamate-rich IDR can interact with the C-terminal positively charged RG-rich region to allow G3BP1 to fold into a compact state. This compact state is an autoinhibitory conformation that disrupts G3BP1 phase separation. However, at a low pH, protonation of the clustered glutamates changes the net charge of the acidic IDR and disrupts its stable electrostatic interactions with the RG-rich region, allowing G3BP1 to expand from its original tightly self-inhibited state and release the RG-rich region. G3BP1 can then further facilitate intermolecular protein–RNA/protein interactions to drive LLPS, which is consistent with the observation that heterotypic interactions among G3BP1 and RNA molecules drive SG assembly [89, 203]. In addition, a low pH can directly trigger Pub1 assembly, and this pH-dependent assembly formation is sensitive to salt concentrations, suggesting that electrostatic interactions promote Pub1 assembly. Self-interactions among the RRM domains are the main drivers for Pub1 phase separation, and acidic pH may change the charge distribution in the RRM domains, thereby mediating the electrostatic interactions [88]. Moreover, the pH range that can induce artificially recombinant ELP phase separation is related to the p*K*a. This suggests to a certain extent that pH can affect protein–solution or protein–protein interactions by changing the number of ionizable residues of proteins, thus triggering phase separation [217]. Likewise, phase separation of Pab1 at low pH is also an electrostatically mediated process [34]; thus, the principle of pH-dependent protein condensate formation mediated by electrostatic interactions may be generalizable to many proteins.

Notably, pH changes not only initiate protein LLPS by facilitating intramolecular or intermolecular interactions but also enhance phase separation and its further maturation into a gel state or a pathological solid state (Fig. 3). Phase separation of α-Syn is mediated by an interplay of electrostatic interactions in the unstructured N-terminal domain and hydrophobic interactions in the central NAC domain, while the charge distribution in these domains is strongly dependent on the pH value. A lower pH serves to change the net charges and hydrophobicity of different domains as well as the interactions between these domains, leading to significant structural reorganization. Thus, pH-mediated diverse changes in α-Syn accelerate the maturation of phase separation and subsequent protein aggregation [149, 209]. Similarly, a reduction in pH can enhance the intramolecular interactions of the phase-separated SARS-CoV-2 N protein and lead to irregularly shaped assemblies with less liquidity, in vitro [205]. Indeed, phase separation proteins that contain flexible LCDs are highly prone to forming pathogenic aggregates. This could explain, to some extent, why hundreds of proteins are highly prone to forming aggregates during aging. During aging or chronic pH stress, these phase separation proteins can transition into irreversible aggregates, which could then lead to persistent condensate formation, such as persistent SG formation, even after the stress subsides. Persistent condensates typically exhibit solid-like properties; and as a consequence, other pathological changes and neurodegenerative disorders occur [233].

### pH changes affect protein solubility

In addition to engaging in the promiscuous interactions that function in phase separation, macromolecules must reach a critical concentration threshold to start LLPS. Evidence indicates that not all LCRs and IDRs function as autonomous modules that drive phase separation; instead, they function as modifier sequences, regulating the solubility of phase-separating proteins and the material properties of condensates [234]. Long-term evolutionary pressure has tuned the solubility of Pub1 to be very close to the critical threshold for phase separation. This not only endows Pub1 with a solubility that is conducive to growth but also enables Pub1 to quickly sense and respond to stress. In fact, changes in pH can affect the solubility of Pub1 and lead to the formation of stress-responsive Pub1 condensates [88]. Changes in pH appear to be able to decrease the solubilities of many proteins. In yeast, a decrease in pH results in a phase transition of cytoplasm from a fluid-like to a solid-like state, which might be caused by decreased solubilities of a series of proteins and subsequent formation of intracellular solid-like assemblies, such as SGs [23, 235]. Moreover, the relationship between pH and protein isoelectric point is closely related to solubility. The closer the pH value is to the isoelectric point of the protein, the lower its solubility, and the more likely it is that phase separation occurs [236, 237]. This could explain to a certain degree why the microtubule-binding repeats of Tau are most prone to phase separation when the pH is close to the isoelectric point but less prone to demixing when the protein solubility is increased in response to pH that is substantially higher or lower than the isoelectric point [16]. Therefore, it is believed that one of the mechanisms by which pH triggers protein phase separation is the alteration of protein solubility (Fig. 3).

### pH changes act as messengers to transmit stress signals

In the face of adverse conditions, intracellular pH might act as a messenger to signal changes in the environment, triggering phase separation of proteins to promote cell fitness. Upon heat shock, cells can integrate signals of different temperatures and temperature-induced pH changes into a unified response to provide a trigger for phase separation. For example, Pab1 undergoes LLPS autonomously through temperature-dependent structural changes under conditions of stressful temperatures [34].

However, how does a cell sense other stresses, such as starvation, to trigger LLPS to help cells survive diverse adverse conditions? Previous studies have indicated that proteins and protein-associated condensates that undergo LLPS under starvation conditions, such as Pub1, Gln1, Dhh1, and PAS, can also respond to low pH [47, 88, 192, 200, 201]. Considering that cytosolic pH is rapidly and reversibly regulated by glucose metabolism, the stress information perceived by these proteins is most likely transmitted through pH. Evidence has shown that cytosolic pH is a second messenger for glucose to mediate activation of the PKA pathway through V-ATPase [43]. Therefore, a change in pH might be an extremely sensitive readout of other changes in the environment, especially starvation, to induce protein LLPS and cellular adaptive responses (Fig. 3).

In this way, pH is capable of playing diverse functional roles in the regulation of LLPS, including by affecting protein–protein/RNA interactions, altering protein solubility, and acting as a messenger to transmit stress signals.

## Conclusion and perspective

From viruses to prokaryotes and eukaryotes, the formation of macromolecular condensates by phase separation is emerging as a principle means for cells to regulate cellular functions and adapt to environmental changes. Cells encounter a variety of stresses, some of which can cause cytoplasmic pH fluctuations. In this review, we have summarized the relationships between pH changes and certain stresses, such as heat shock, nutrient stress, and osmotic stress, and described which proteins or physiological processes can respond to stress-associated pH changes through phase separation. We have also highlighted the diverse ways by which pH fluctuation can influence protein phase separation. For example, pH can act as a signal to transmit stress information, mediate protein–protein/RNA interactions, and affect protein solubility, thereby regulating protein/RNA phase separation.

Despite the research progress concerning the relationships between stress-associated pH changes and phase separation discussed in this review, further in-depth investigations are still needed. It is worth noting that pH might not be the sole determinant of stress-induced phase separation and condensate formation. For stresses such as heat shock, changes in both intracellular temperature and pH are involved, which can lead them to differences in protein phase separation behavior and condensate material properties [88]. The interplay of pH, temperature, ion strength, RNA concentration, protein concentration and other factors forms a sophisticated network that dynamically affects the phase behavior of proteins. However, some questions remain. How does pH interact with other factors in this process? What are the differences and similarities in the roles of pH among the different stress-induced phase separation processes? Preliminary evidence suggests that the properties of different condensate materials formed by different groups of proteins can be used by cells to build a hierarchical stress-adaptive system that is fine-tuned to different conditions [88]. In other words, when encountering different types of stresses or the same stress with different intensity or duration, a cell can regulate the activities of multiple proteins to achieve specific biological functions by concentrating specific cellular components in the condensates (or excluding them from the condensates) for a favorable period of time. The cells can then determine when to restart growth. In this way, control of condensates can be used by the cells as a method to promote adaptation to stress. Therefore, revealing the differences and similarities among the various roles of pH in addressing different types of stress will provide insights into the mechanisms underlying the protein separation involved in cellular adaptation. Moreover, pH values might be changed considerably by different stresses, and a given protein might display different phase separation behaviors under different pH values. Therefore, the identification of proteins that respond to different pH values or have behavior changes that accompany pH changes may provide vital clues for investigation of the machineries involved in the influences of pH on cellular functions.

Finally, intracellular pH changes and phase separation condensate formation are linked to aging, aging-related neurodegenerative diseases, and cancers. It would be interesting to further investigate how aging-induced pH changes affect protein phase separation. Importantly, innovative drug delivery strategies could be developed for specific local cancer therapy by exploiting the altered intracellular and extracellular pH in tumors. Attempts to modulate pH and SG formation could also spur the development of innovative approaches for cancer therapy.

## Declarations

### Data availability

Data sharing not applicable to this article as no datasets were generated or analyzed during the current study.
